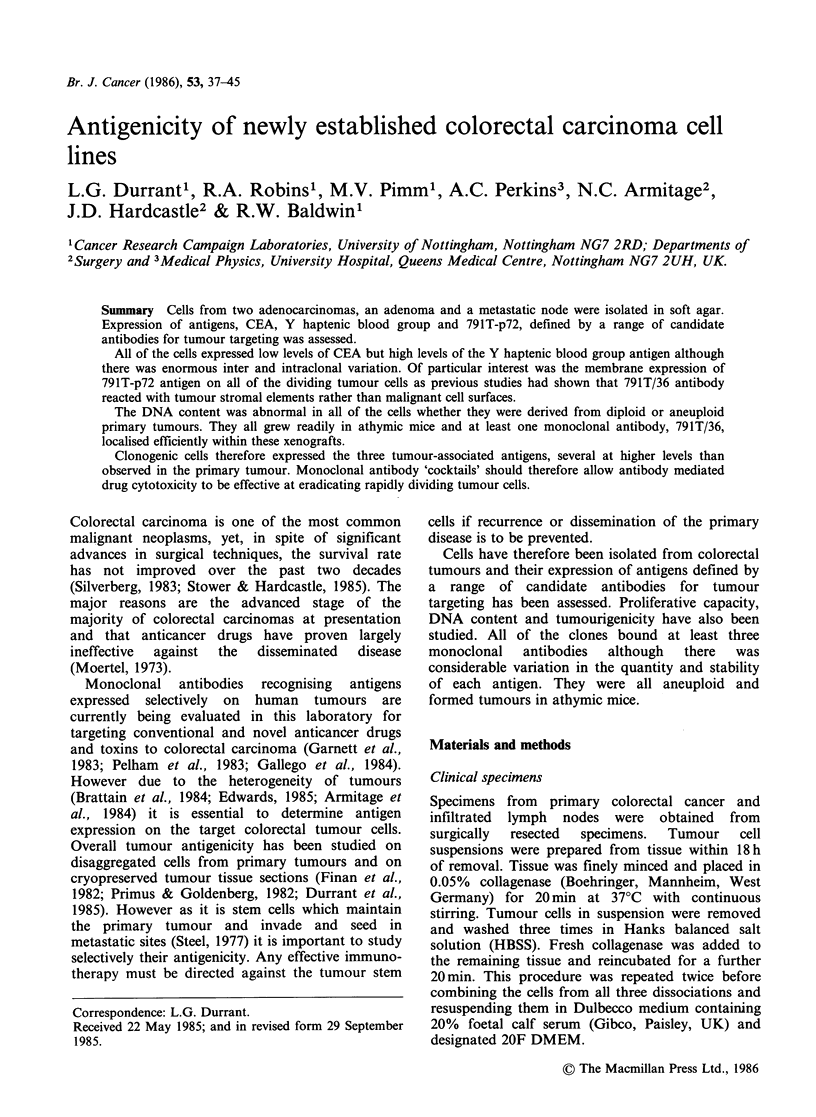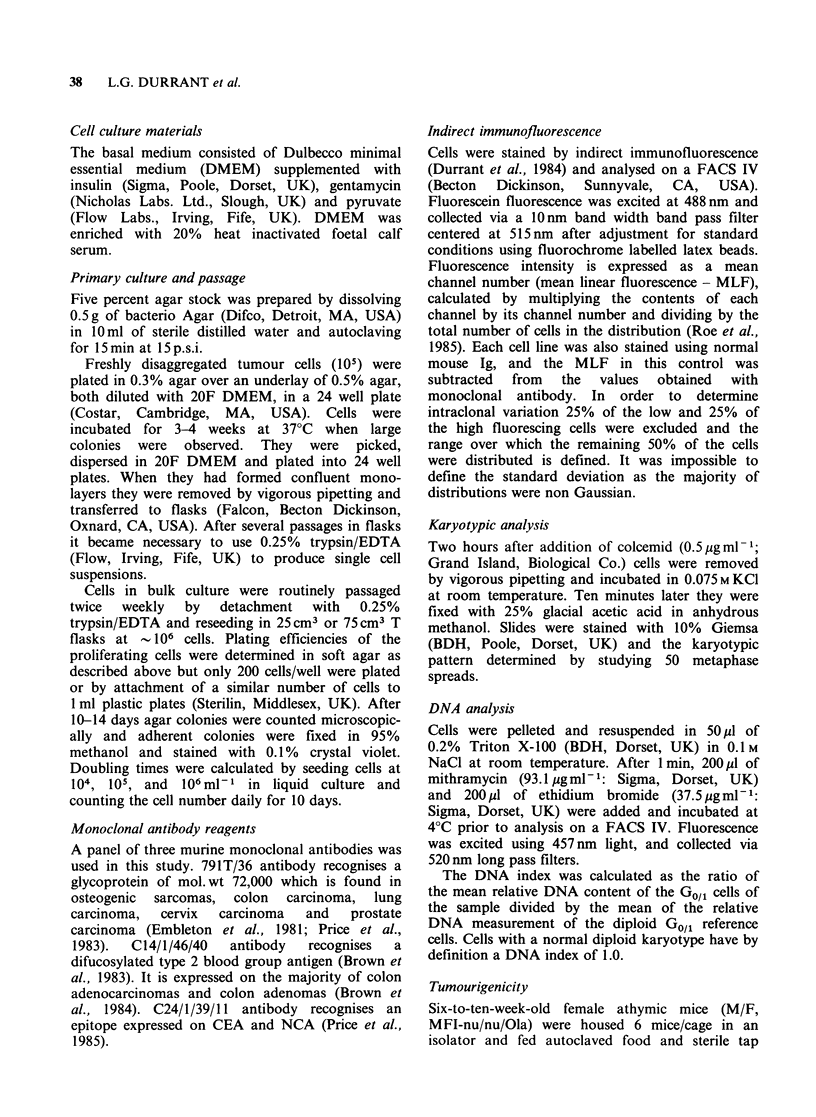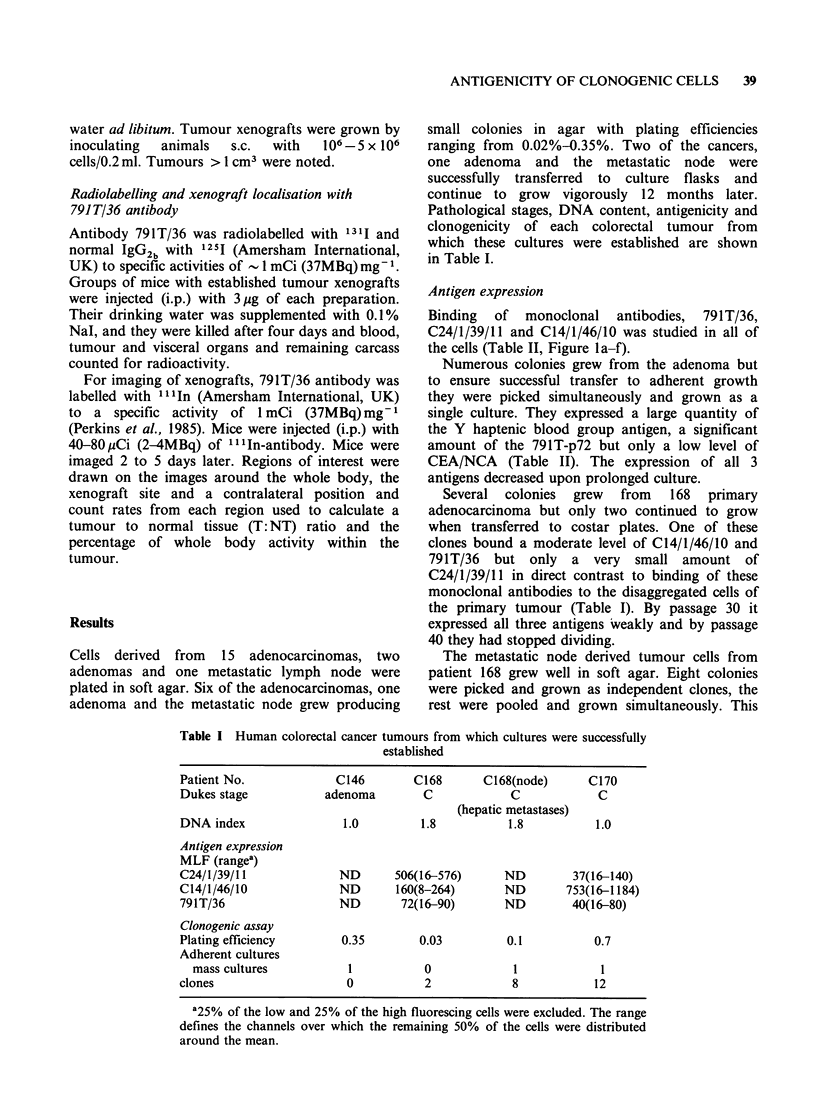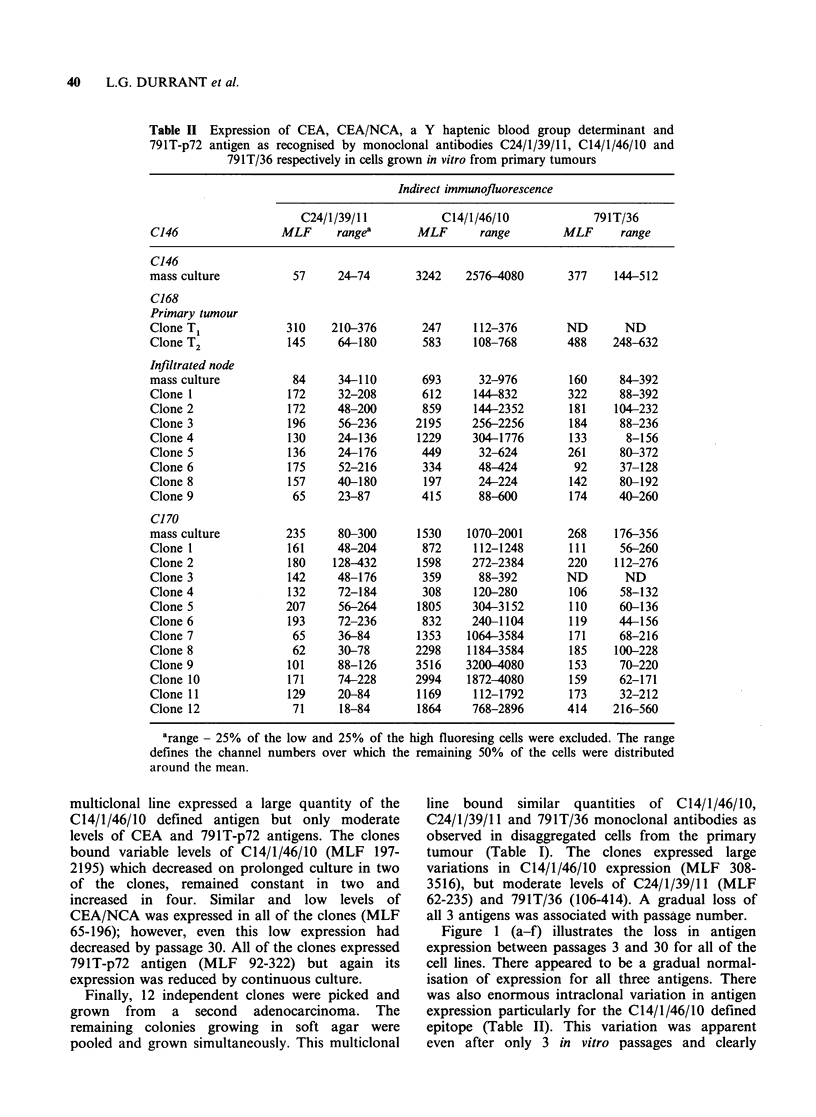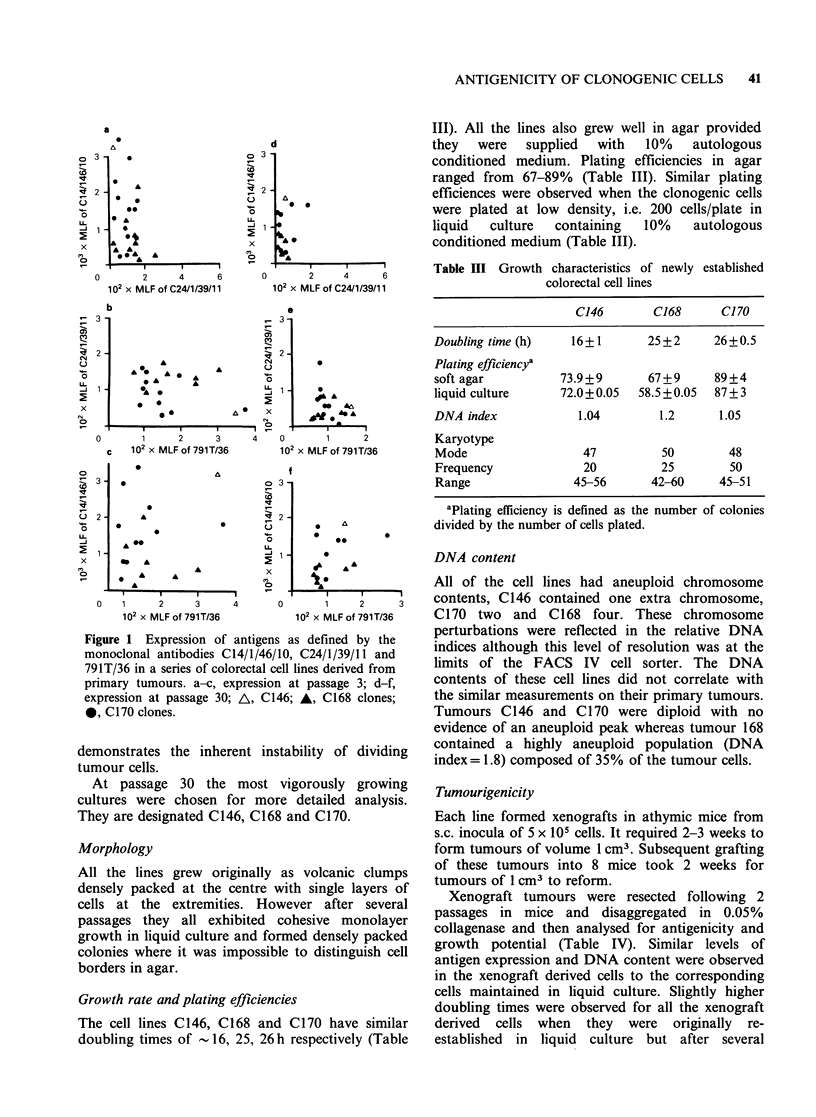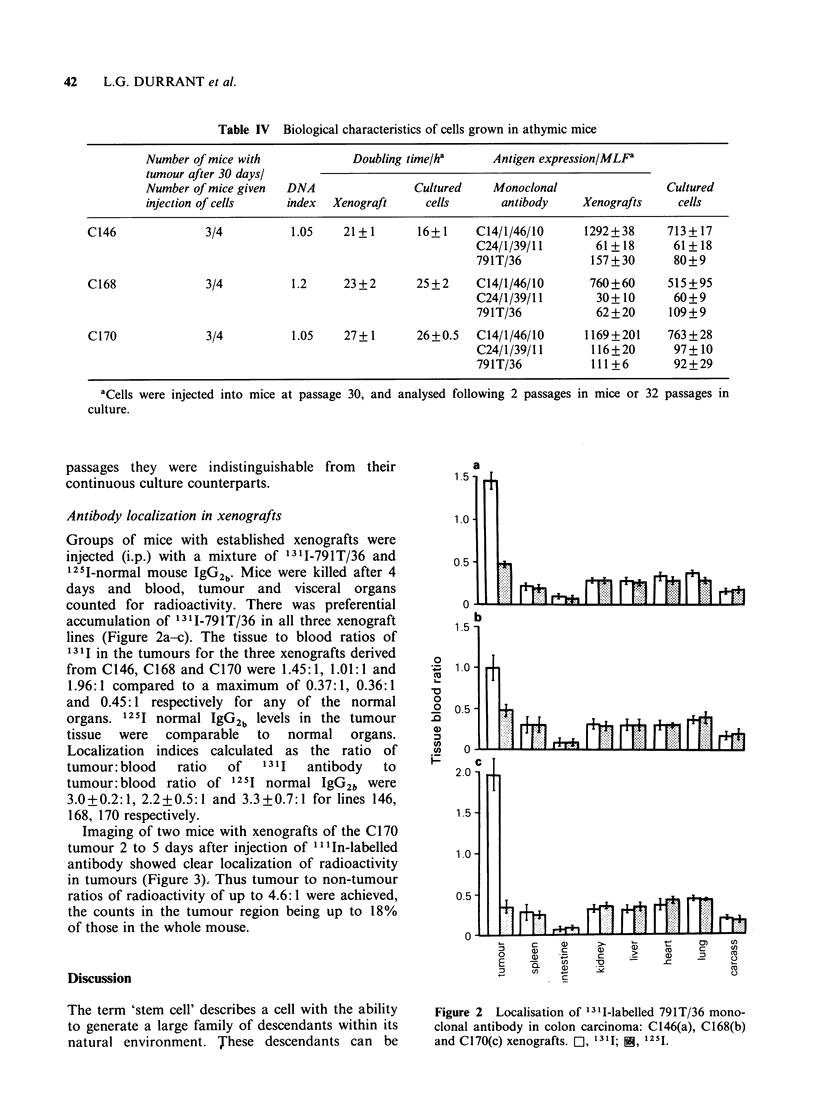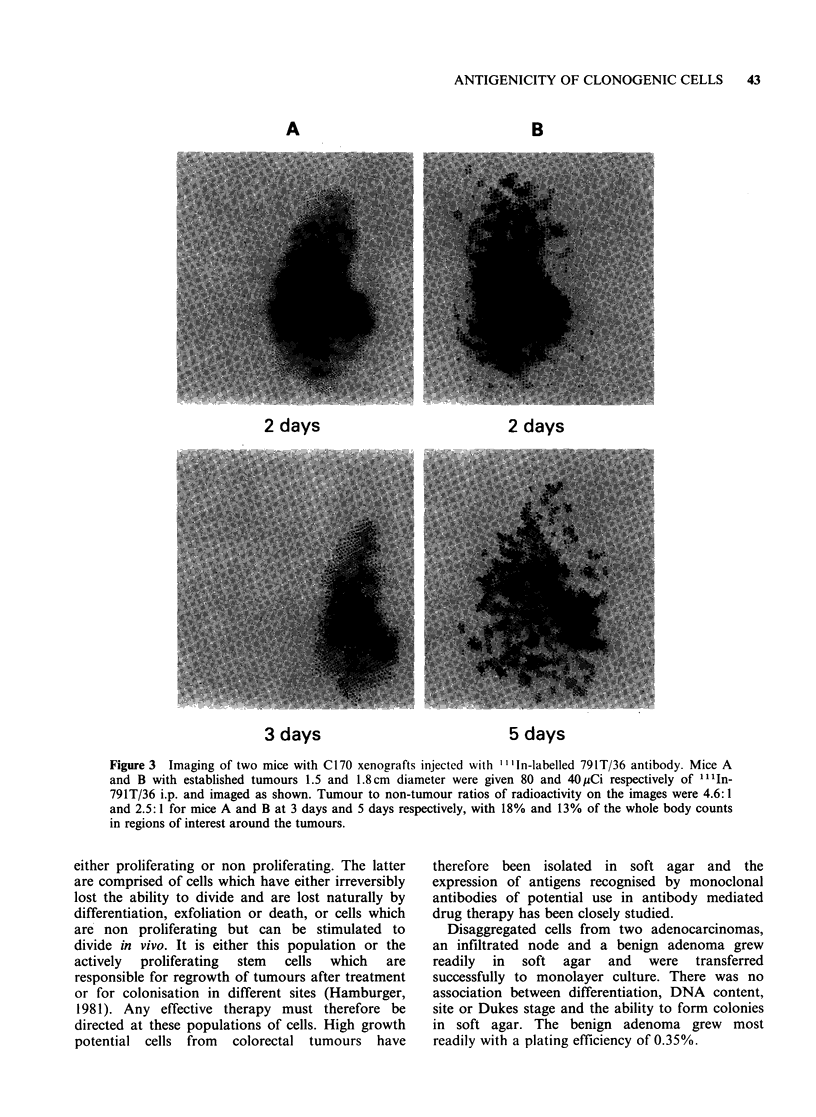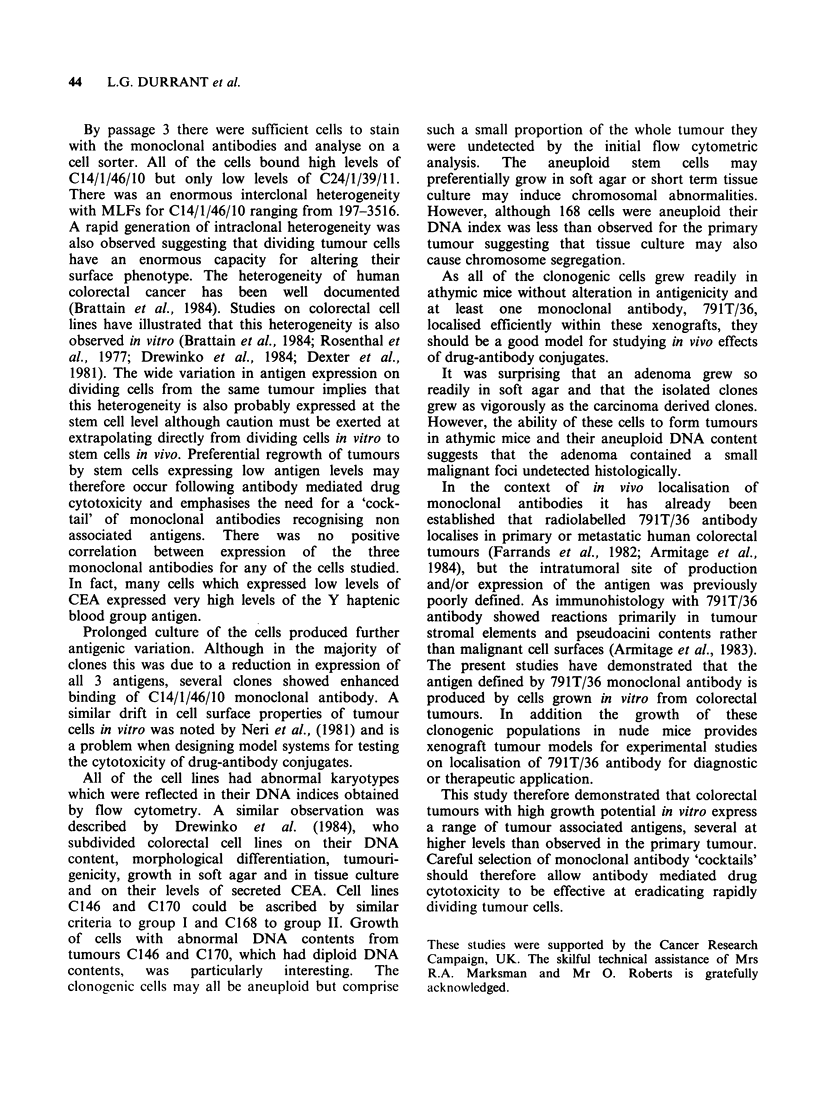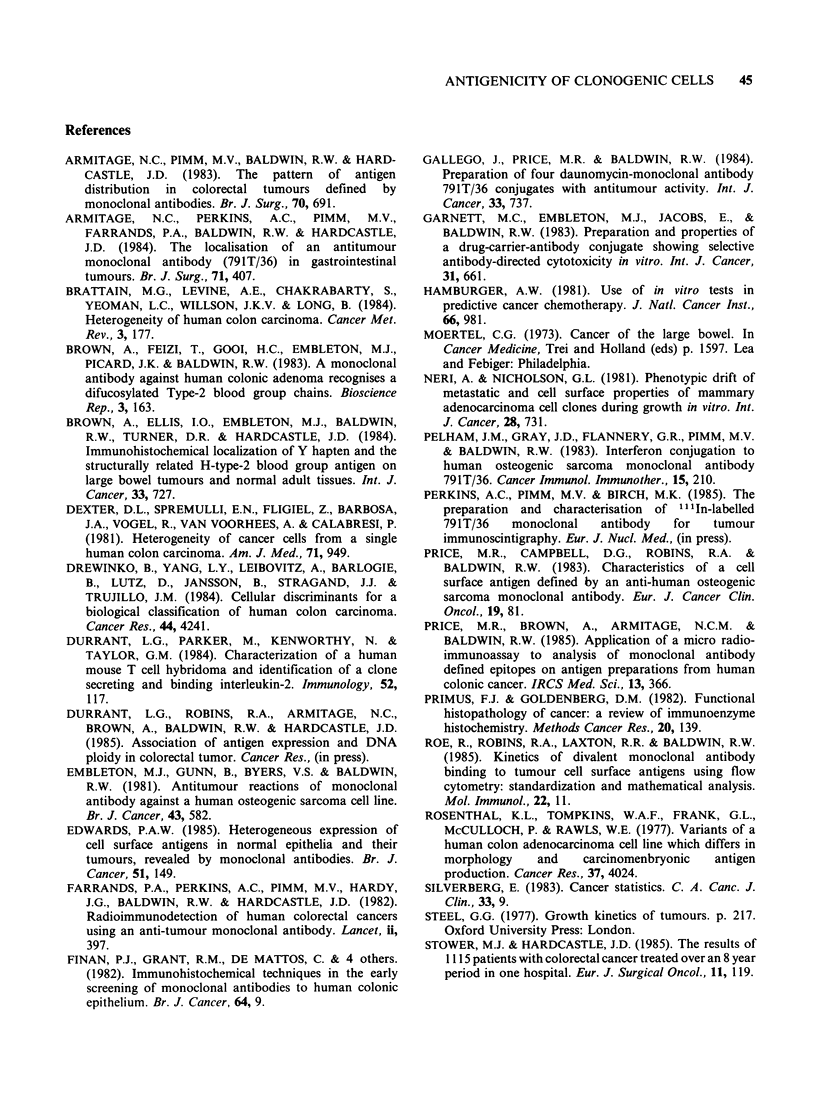# Antigenicity of newly established colorectal carcinoma cell lines.

**DOI:** 10.1038/bjc.1986.6

**Published:** 1986-01

**Authors:** L. G. Durrant, R. A. Robins, M. V. Pimm, A. C. Perkins, N. C. Armitage, J. D. Hardcastle, R. W. Baldwin

## Abstract

**Images:**


Br. J. Cancer (1986), 53, 37–45

### Antigenicity of newly established colorectal carcinoma cell lines

L.G. Durrant[1], R.A. Robins[1], M.V. Pimm[1], A.C. Perkins[3], N.C. Armitage[2], J.D. Hardcastle[2] & R.W. Baldwin[1]

[1]*Cancer Research Campaign Laboratories, University of Nottingham, Nottingham NG7 2RD; Departments of* [2]*Surgery and* [3]*Medical Physics, University Hospital, Queens Medical Centre, Nottingham NG7 2UH, UK.*

**Summary** Cells from two adenocarcinomas, an adenoma and a metastatic node were isolated in soft agar. Expression of antigens, CEA, Y haptenic blood group and 791T-p72, defined by a range of candidate antibodies for tumour targeting was assessed.

All of the cells expressed low levels of CEA but high levels of the Y haptenic blood group antigen although there was enormous inter and intraclonal variation. Of particular interest was the membrane expression of 791T-p72 antigen on all of the dividing tumour cells as previous studies had shown that 791T/36 antibody reacted with tumour stromal elements rather than malignant cell surfaces.

The DNA content was abnormal in all of the cells whether they were derived from diploid or aneuploid primary tumours. They all grew readily in athymic mice and at least one monoclonal antibody, 791T/36, localised efficiently within these xenografts.

Clonogenic cells therefore expressed the three tumour-associated antigens, several at higher levels than observed in the primary tumour. Monoclonal antibody 'cocktails' should therefore allow antibody mediated drug cytotoxicity to be effective at eradicating rapidly dividing tumour cells.

Colorectal carcinoma is one of the most common malignant neoplasms, yet, in spite of significant advances in surgical techniques, the survival rate has not improved over the past two decades (Silverberg, 1983; Stower & Hardcastle, 1985). The major reasons are the advanced stage of the majority of colorectal carcinomas at presentation and that anticancer drugs have proven largely ineffective against the disseminated disease (Moertel, 1973).

Monoclonal antibodies recognising antigens expressed selectively on human tumours are currently being evaluated in this laboratory for targeting conventional and novel anticancer drugs and toxins to colorectal carcinoma (Garnett *et al.*, 1983; Pelham *et al.*, 1983; Gallego *et al.*, 1984). However due to the heterogeneity of tumours (Brattain *et al.*, 1984; Edwards, 1985; Armitage *et al.*, 1984) it is essential to determine antigen expression on the target colorectal tumour cells. Overall tumour antigenicity has been studied on disaggregated cells from primary tumours and on cryopreserved tumour tissue sections (Finan *et al.*, 1982; Primus & Goldenberg, 1982; Durrant *et al.*, 1985). However as it is stem cells which maintain the primary tumour and invade and seed in metastatic sites (Steel, 1977) it is important to study selectively their antigenicity. Any effective immunotherapy must be directed against the tumour stem cells if recurrence or dissemination of the primary disease is to be prevented.

Cells have therefore been isolated from colorectal tumours and their expression of antigens defined by a range of candidate antibodies for tumour targeting has been assessed. Proliferative capacity, DNA content and tumourigenicity have also been studied. All of the clones bound at least three monoclonal antibodies although there was considerable variation in the quantity and stability of each antigen. They were all aneuploid and formed tumours in athymic mice.

### Materials and methods

### *Clinical specimens*

Specimens from primary colorectal cancer and infiltrated lymph nodes were obtained from surgically resected specimens. Tumour cell suspensions were prepared from tissue within 18 h of removal. Tissue was finely minced and placed in 0.05% collagenase (Boehringer, Mannheim, West Germany) for 20 min at 37°C with continuous stirring. Tumour cells in suspension were removed and washed three times in Hanks balanced salt solution (HBSS). Fresh collagenase was added to the remaining tissue and reincubated for a further 20 min. This procedure was repeated twice before combining the cells from all three dissociations and resuspending them in Dulbecco medium containing 20% foetal calf serum (Gibco, Paisley, UK) and designated 20F DMEM.

Correspondence: L.G. Durrant.
Received 22 May 1985; and in revised form 29 September 1985.

© The Macmillan Press Ltd., 1986

*Cell culture materials*

The basal medium consisted of Dulbecco minimal essential medium (DMEM) supplemented with insulin (Sigma, Poole, Dorset, UK), gentamycin (Nicholas Labs. Ltd., Slough, UK) and pyruvate (Flow Labs., Irving, Fife, UK). DMEM was enriched with 20% heat inactivated foetal calf serum.

*Primary culture and passage*

Five percent agar stock was prepared by dissolving 0.5 g of bacterio Agar (Difco, Detroit, MA, USA) in 10 ml of sterile distilled water and autoclaving for 15 min at 15 p.s.i.

Freshly disaggregated tumour cells ($10^5$) were plated in 0.3% agar over an underlay of 0.5% agar, both diluted with 20F DMEM, in a 24 well plate (Costar, Cambridge, MA, USA). Cells were incubated for 3–4 weeks at 37°C when large colonies were observed. They were picked, dispersed in 20F DMEM and plated into 24 well plates. When they had formed confluent monolayers they were removed by vigorous pipetting and transferred to flasks (Falcon, Becton Dickinson, Oxnard, CA, USA). After several passages in flasks it became necessary to use 0.25% trypsin/EDTA (Flow, Irving, Fife, UK) to produce single cell suspensions.

Cells in bulk culture were routinely passaged twice weekly by detachment with 0.25% trypsin/EDTA and reseeding in 25 $cm^3$ or 75 $cm^3$ T flasks at $\sim 10^6$ cells. Plating efficiencies of the proliferating cells were determined in soft agar as described above but only 200 cells/well were plated or by attachment of a similar number of cells to 1 ml plastic plates (Sterilin, Middlesex, UK). After 10–14 days agar colonies were counted microscopically and adherent colonies were fixed in 95% methanol and stained with 0.1% crystal violet. Doubling times were calculated by seeding cells at $10^4$, $10^5$, and $10^6 ml^{-1}$ in liquid culture and counting the cell number daily for 10 days.

*Monoclonal antibody reagents*

A panel of three murine monoclonal antibodies was used in this study. 791T/36 antibody recognises a glycoprotein of mol. wt 72,000 which is found in osteogenic sarcomas, colon carcinoma, lung carcinoma, cervix carcinoma and prostate carcinoma (Embleton *et al.*, 1981; Price *et al.*, 1983). C14/1/46/40 antibody recognises a difucosylated type 2 blood group antigen (Brown *et al.*, 1983). It is expressed on the majority of colon adenocarcinomas and colon adenomas (Brown *et al.*, 1984). C24/1/39/11 antibody recognises an epitope expressed on CEA and NCA (Price *et al.*, 1985).

*Indirect immunofluorescence*

Cells were stained by indirect immunofluorescence (Durrant *et al.*, 1984) and analysed on a FACS IV (Becton Dickinson, Sunnyvale, CA, USA). Fluorescein fluorescence was excited at 488 nm and collected via a 10 nm band width band pass filter centered at 515 nm after adjustment for standard conditions using fluorochrome labelled latex beads. Fluorescence intensity is expressed as a mean channel number (mean linear fluorescence – MLF), calculated by multiplying the contents of each channel by its channel number and dividing by the total number of cells in the distribution (Roe *et al.*, 1985). Each cell line was also stained using normal mouse Ig, and the MLF in this control was subtracted from the values obtained with monoclonal antibody. In order to determine intraclonal variation 25% of the low and 25% of the high fluorescing cells were excluded and the range over which the remaining 50% of the cells were distributed is defined. It was impossible to define the standard deviation as the majority of distributions were non Gaussian.

*Karyotypic analysis*

Two hours after addition of colcemid ($0.5 \mu g ml^{-1}$; Grand Island, Biological Co.) cells were removed by vigorous pipetting and incubated in 0.075 M KCl at room temperature. Ten minutes later they were fixed with 25% glacial acetic acid in anhydrous methanol. Slides were stained with 10% Giemsa (BDH, Poole, Dorset, UK) and the karyotypic pattern determined by studying 50 metaphase spreads.

*DNA analysis*

Cells were pelleted and resuspended in 50 $\mu l$ of 0.2% Triton X-100 (BDH, Dorset, UK) in 0.1 M NaCl at room temperature. After 1 min, 200 $\mu l$ of mithramycin ($93.1 \mu g ml^{-1}$: Sigma, Dorset, UK) and 200 $\mu l$ of ethidium bromide ($37.5 \mu g ml^{-1}$: Sigma, Dorset, UK) were added and incubated at 4°C prior to analysis on a FACS IV. Fluorescence was excited using 457 nm light, and collected via 520 nm long pass filters.

The DNA index was calculated as the ratio of the mean relative DNA content of the $G_{0/1}$ cells of the sample divided by the mean of the relative DNA measurement of the diploid $G_{0/1}$ reference cells. Cells with a normal diploid karyotype have by definition a DNA index of 1.0.

*Tumourigenicity*

Six-to-ten-week-old female athymic mice (M/F, MFI-nu/nu/Ola) were housed 6 mice/cage in an isolator and fed autoclaved food and sterile tap water *ad libitum*. Tumour xenografts were grown by inoculating animals s.c. with $10^6-5\times10^6$ cells/0.2 ml. Tumours $>1\,cm^3$ were noted.

*Radiolabelling and xenograft localisation with 791T/36 antibody*

Antibody 791T/36 was radiolabelled with $^{131}I$ and normal $IgG_{2b}$ with $^{125}I$ (Amersham International, UK) to specific activities of $\sim 1$ mCi (37MBq) $mg^{-1}$. Groups of mice with established tumour xenografts were injected (i.p.) with 3 $\mu g$ of each preparation. Their drinking water was supplemented with 0.1% NaI, and they were killed after four days and blood, tumour and visceral organs and remaining carcass counted for radioactivity.

For imaging of xenografts, 791T/36 antibody was labelled with $^{111}In$ (Amersham International, UK) to a specific activity of 1 mCi (37MBq) $mg^{-1}$ (Perkins *et al.*, 1985). Mice were injected (i.p.) with 40–80 $\mu$Ci (2–4MBq) of $^{111}In$-antibody. Mice were imaged 2 to 5 days later. Regions of interest were drawn on the images around the whole body, the xenograft site and a contralateral position and count rates from each region used to calculate a tumour to normal tissue (T:NT) ratio and the percentage of whole body activity within the tumour.

### Results

Cells derived from 15 adenocarcinomas, two adenomas and one metastatic lymph node were plated in soft agar. Six of the adenocarcinomas, one adenoma and the metastatic node grew producing small colonies in agar with plating efficiencies ranging from 0.02%–0.35%. Two of the cancers, one adenoma and the metastatic node were successfully transferred to culture flasks and continue to grow vigorously 12 months later. Pathological stages, DNA content, antigenicity and clonogenicity of each colorectal tumour from which these cultures were established are shown in Table I.

*Antigen expression*

Binding of monoclonal antibodies, 791T/36, C24/1/39/11 and C14/1/46/10 was studied in all of the cells (Table II, Figure 1a–f).

Numerous colonies grew from the adenoma but to ensure successful transfer to adherent growth they were picked simultaneously and grown as a single culture. They expressed a large quantity of the Y haptenic blood group antigen, a significant amount of the 791T-p72 but only a low level of CEA/NCA (Table II). The expression of all 3 antigens decreased upon prolonged culture.

Several colonies grew from 168 primary adenocarcinoma but only two continued to grow when transferred to costar plates. One of these clones bound a moderate level of C14/1/46/10 and 791T/36 but only a very small amount of C24/1/39/11 in direct contrast to binding of these monoclonal antibodies to the disaggregated cells of the primary tumour (Table I). By passage 30 it expressed all three antigens weakly and by passage 40 they had stopped dividing.

The metastatic node derived tumour cells from patient 168 grew well in soft agar. Eight colonies were picked and grown as independent clones, the rest were pooled and grown simultaneously. This

**Table I** Human colorectal cancer tumours from which cultures were successfully established

| Patient No. | C146 | C168 | C168(node) | C170 |
|---|---|---|---|---|
| Dukes stage | adenoma | C | C (hepatic metastases) | C |
| DNA index | 1.0 | 1.8 | 1.8 | 1.0 |
| *Antigen expression* | | | | |
| MLF (range[a]) | | | | |
| C24/1/39/11 | ND | 506(16–576) | ND | 37(16–140) |
| C14/1/46/10 | ND | 160(8–264) | ND | 753(16–1184) |
| 791T/36 | ND | 72(16–90) | ND | 40(16–80) |
| *Clonogenic assay* | | | | |
| Plating efficiency | 0.35 | 0.03 | 0.1 | 0.7 |
| Adherent cultures | | | | |
| mass cultures | 1 | 0 | 1 | 1 |
| clones | 0 | 2 | 8 | 12 |

[a]25% of the low and 25% of the high fluorescing cells were excluded. The range defines the channels over which the remaining 50% of the cells were distributed around the mean.

**Table II** Expression of CEA, CEA/NCA, a Y haptenic blood group determinant and 791T-p72 antigen as recognised by monoclonal antibodies C24/1/39/11, C14/1/46/10 and 791T/36 respectively in cells grown *in vitro* from primary tumours

| | *Indirect immunofluorescence* | | | | | |
|---|---|---|---|---|---|---|
| | C24/1/39/11 | | C14/1/46/10 | | 791T/36 | |
| *C146* | *MLF* | *range*[a] | *MLF* | *range* | *MLF* | *range* |
| *C146* | | | | | | |
| mass culture | 57 | 24–74 | 3242 | 2576–4080 | 377 | 144–512 |
| *C168* | | | | | | |
| *Primary tumour* | | | | | | |
| Clone $T_1$ | 310 | 210–376 | 247 | 112–376 | ND | ND |
| Clone $T_2$ | 145 | 64–180 | 583 | 108–768 | 488 | 248–632 |
| *Infiltrated node* | | | | | | |
| mass culture | 84 | 34–110 | 693 | 32–976 | 160 | 84–392 |
| Clone 1 | 172 | 32–208 | 612 | 144–832 | 322 | 88–392 |
| Clone 2 | 172 | 48–200 | 859 | 144–2352 | 181 | 104–232 |
| Clone 3 | 196 | 56–236 | 2195 | 256–2256 | 184 | 88–236 |
| Clone 4 | 130 | 24–136 | 1229 | 304–1776 | 133 | 8–156 |
| Clone 5 | 136 | 24–176 | 449 | 32–624 | 261 | 80–372 |
| Clone 6 | 175 | 52–216 | 334 | 48–424 | 92 | 37–128 |
| Clone 8 | 157 | 40–180 | 197 | 24–224 | 142 | 80–192 |
| Clone 9 | 65 | 23–87 | 415 | 88–600 | 174 | 40–260 |
| *C170* | | | | | | |
| mass culture | 235 | 80–300 | 1530 | 1070–2001 | 268 | 176–356 |
| Clone 1 | 161 | 48–204 | 872 | 112–1248 | 111 | 56–260 |
| Clone 2 | 180 | 128–432 | 1598 | 272–2384 | 220 | 112–276 |
| Clone 3 | 142 | 48–176 | 359 | 88–392 | ND | ND |
| Clone 4 | 132 | 72–184 | 308 | 120–280 | 106 | 58–132 |
| Clone 5 | 207 | 56–264 | 1805 | 304–3152 | 110 | 60–136 |
| Clone 6 | 193 | 72–236 | 832 | 240–1104 | 119 | 44–156 |
| Clone 7 | 65 | 36–84 | 1353 | 1064–3584 | 171 | 68–216 |
| Clone 8 | 62 | 30–78 | 2298 | 1184–3584 | 185 | 100–228 |
| Clone 9 | 101 | 88–126 | 3516 | 3200–4080 | 153 | 70–220 |
| Clone 10 | 171 | 74–228 | 2994 | 1872–4080 | 159 | 62–171 |
| Clone 11 | 129 | 20–84 | 1169 | 112–1792 | 173 | 32–212 |
| Clone 12 | 71 | 18–84 | 1864 | 768–2896 | 414 | 216–560 |

[a]range – 25% of the low and 25% of the high fluoresing cells were excluded. The range defines the channel numbers over which the remaining 50% of the cells were distributed around the mean.

multiclonal line expressed a large quantity of the C14/1/46/10 defined antigen but only moderate levels of CEA and 791T-p72 antigens. The clones bound variable levels of C14/1/46/10 (MLF 197-2195) which decreased on prolonged culture in two of the clones, remained constant in two and increased in four. Similar and low levels of CEA/NCA was expressed in all of the clones (MLF 65-196); however, even this low expression had decreased by passage 30. All of the clones expressed 791T-p72 antigen (MLF 92-322) but again its expression was reduced by continuous culture.

Finally, 12 independent clones were picked and grown from a second adenocarcinoma. The remaining colonies growing in soft agar were pooled and grown simultaneously. This multiclonal line bound similar quantities of C14/1/46/10, C24/1/39/11 and 791T/36 monoclonal antibodies as observed in disaggregated cells from the primary tumour (Table I). The clones expressed large variations in C14/1/46/10 expression (MLF 308-3516), but moderate levels of C24/1/39/11 (MLF 62-235) and 791T/36 (106-414). A gradual loss of all 3 antigens was associated with passage number.

Figure 1 (a–f) illustrates the loss in antigen expression between passages 3 and 30 for all of the cell lines. There appeared to be a gradual normalisation of expression for all three antigens. There was also enormous intraclonal variation in antigen expression particularly for the C14/1/46/10 defined epitope (Table II). This variation was apparent even after only 3 *in vitro* passages and clearly

**Figure 1** Expression of antigens as defined by the monoclonal antibodies C14/1/46/10, C24/1/39/11 and 791T/36 in a series of colorectal cell lines derived from primary tumours. a–c, expression at passage 3; d–f, expression at passage 30; △, C146; ▲, C168 clones; ●, C170 clones.

demonstrates the inherent instability of dividing tumour cells.

At passage 30 the most vigorously growing cultures were chosen for more detailed analysis. They are designated C146, C168 and C170.

### *Morphology*

All the lines grew originally as volcanic clumps densely packed at the centre with single layers of cells at the extremities. However after several passages they all exhibited cohesive monolayer growth in liquid culture and formed densely packed colonies where it was impossible to distinguish cell borders in agar.

### *Growth rate and plating efficiencies*

The cell lines C146, C168 and C170 have similar doubling times of ~16, 25, 26 h respectively (Table III). All the lines also grew well in agar provided they were supplied with 10% autologous conditioned medium. Plating efficiencies in agar ranged from 67–89% (Table III). Similar plating efficiences were observed when the clonogenic cells were plated at low density, i.e. 200 cells/plate in liquid culture containing 10% autologous conditioned medium (Table III).

**Table III** Growth characteristics of newly established colorectal cell lines

| | *C146* | *C168* | *C170* |
|---|---|---|---|
| *Doubling time* (h) | 16±1 | 25±2 | 26±0.5 |
| *Plating efficiency*[a] | | | |
| soft agar | 73.9±9 | 67±9 | 89±4 |
| liquid culture | 72.0±0.05 | 58.5±0.05 | 87±3 |
| *DNA index* | 1.04 | 1.2 | 1.05 |
| Karyotype | | | |
| Mode | 47 | 50 | 48 |
| Frequency | 20 | 25 | 50 |
| Range | 45–56 | 42–60 | 45–51 |

[a]Plating efficiency is defined as the number of colonies divided by the number of cells plated.

### *DNA content*

All of the cell lines had aneuploid chromosome contents, C146 contained one extra chromosome, C170 two and C168 four. These chromosome perturbations were reflected in the relative DNA indices although this level of resolution was at the limits of the FACS IV cell sorter. The DNA contents of these cell lines did not correlate with the similar measurements on their primary tumours. Tumours C146 and C170 were diploid with no evidence of an aneuploid peak whereas tumour 168 contained a highly aneuploid population (DNA index = 1.8) composed of 35% of the tumour cells.

### *Tumourigenicity*

Each line formed xenografts in athymic mice from s.c. inocula of $5 \times 10^5$ cells. It required 2–3 weeks to form tumours of volume 1 $cm^3$. Subsequent grafting of these tumours into 8 mice took 2 weeks for tumours of 1 $cm^3$ to reform.

Xenograft tumours were resected following 2 passages in mice and disaggregated in 0.05% collagenase and then analysed for antigenicity and growth potential (Table IV). Similar levels of antigen expression and DNA content were observed in the xenograft derived cells to the corresponding cells maintained in liquid culture. Slightly higher doubling times were observed for all the xenograft derived cells when they were originally re-established in liquid culture but after several

**Table IV** Biological characteristics of cells grown in athymic mice

| | *Number of mice with tumour after 30 days/ Number of mice given injection of cells* | *DNA index* | *Doubling time/h*[a] | | *Antigen expression/MLF*[a] | | |
|---|---|---|---|---|---|---|---|
| | | | *Xenograft* | *Cultured cells* | *Monoclonal antibody* | *Xenografts* | *Cultured cells* |
| C146 | 3/4 | 1.05 | 21±1 | 16±1 | C14/1/46/10 | 1292±38 | 713±17 |
| | | | | | C24/1/39/11 | 61±18 | 61±18 |
| | | | | | 791T/36 | 157±30 | 80±9 |
| C168 | 3/4 | 1.2 | 23±2 | 25±2 | C14/1/46/10 | 760±60 | 515±95 |
| | | | | | C24/1/39/11 | 30±10 | 60±9 |
| | | | | | 791T/36 | 62±20 | 109±9 |
| C170 | 3/4 | 1.05 | 27±1 | 26±0.5 | C14/1/46/10 | 1169±201 | 763±28 |
| | | | | | C24/1/39/11 | 116±20 | 97±10 |
| | | | | | 791T/36 | 111±6 | 92±29 |

[a]Cells were injected into mice at passage 30, and analysed following 2 passages in mice or 32 passages in culture.

passages they were indistinguishable from their continuous culture counterparts.

*Antibody localization in xenografts*

Groups of mice with established xenografts were injected (i.p.) with a mixture of $^{131}$I-791T/36 and $^{125}$I-normal mouse $IgG_{2b}$. Mice were killed after 4 days and blood, tumour and visceral organs counted for radioactivity. There was preferential accumulation of $^{131}$I-791T/36 in all three xenograft lines (Figure 2a–c). The tissue to blood ratios of $^{131}$I in the tumours for the three xenografts derived from C146, C168 and C170 were 1.45:1, 1.01:1 and 1.96:1 compared to a maximum of 0.37:1, 0.36:1 and 0.45:1 respectively for any of the normal organs. $^{125}$I normal $IgG_{2b}$ levels in the tumour tissue were comparable to normal organs. Localization indices calculated as the ratio of tumour:blood ratio of $^{131}$I antibody to tumour:blood ratio of $^{125}$I normal $IgG_{2b}$ were 3.0±0.2:1, 2.2±0.5:1 and 3.3±0.7:1 for lines 146, 168, 170 respectively.

Imaging of two mice with xenografts of the C170 tumour 2 to 5 days after injection of $^{111}$In-labelled antibody showed clear localization of radioactivity in tumours (Figure 3). Thus tumour to non-tumour ratios of radioactivity of up to 4.6:1 were achieved, the counts in the tumour region being up to 18% of those in the whole mouse.

**Figure 2** Localisation of $^{131}$I-labelled 791T/36 monoclonal antibody in colon carcinoma: C146(a), C168(b) and C170(c) xenografts. □, $^{131}$I; ▩, $^{125}$I.

### Discussion

The term 'stem cell' describes a cell with the ability to generate a large family of descendants within its natural environment. These descendants can be

**Figure 3** Imaging of two mice with C170 xenografts injected with $^{111}$In-labelled 791T/36 antibody. Mice A and B with established tumours 1.5 and 1.8 cm diameter were given 80 and 40 $\mu$Ci respectively of $^{111}$In-791T/36 i.p. and imaged as shown. Tumour to non-tumour ratios of radioactivity on the images were 4.6:1 and 2.5:1 for mice A and B at 3 days and 5 days respectively, with 18% and 13% of the whole body counts in regions of interest around the tumours.

either proliferating or non proliferating. The latter are comprised of cells which have either irreversibly lost the ability to divide and are lost naturally by differentiation, exfoliation or death, or cells which are non proliferating but can be stimulated to divide *in vivo*. It is either this population or the actively proliferating stem cells which are responsible for regrowth of tumours after treatment or for colonisation in different sites (Hamburger, 1981). Any effective therapy must therefore be directed at these populations of cells. High growth potential cells from colorectal tumours have therefore been isolated in soft agar and the expression of antigens recognised by monoclonal antibodies of potential use in antibody mediated drug therapy has been closely studied.

Disaggregated cells from two adenocarcinomas, an infiltrated node and a benign adenoma grew readily in soft agar and were transferred successfully to monolayer culture. There was no association between differentiation, DNA content, site or Dukes stage and the ability to form colonies in soft agar. The benign adenoma grew most readily with a plating efficiency of 0.35%.

By passage 3 there were sufficient cells to stain with the monoclonal antibodies and analyse on a cell sorter. All of the cells bound high levels of C14/1/46/10 but only low levels of C24/1/39/11. There was an enormous interclonal heterogeneity with MLFs for C14/1/46/10 ranging from 197–3516. A rapid generation of intraclonal heterogeneity was also observed suggesting that dividing tumour cells have an enormous capacity for altering their surface phenotype. The heterogeneity of human colorectal cancer has been well documented (Brattain *et al.*, 1984). Studies on colorectal cell lines have illustrated that this heterogeneity is also observed *in vitro* (Brattain *et al.*, 1984; Rosenthal *et al.*, 1977; Drewinko *et al.*, 1984; Dexter *et al.*, 1981). The wide variation in antigen expression on dividing cells from the same tumour implies that this heterogeneity is also probably expressed at the stem cell level although caution must be exerted at extrapolating directly from dividing cells *in vitro* to stem cells *in vivo*. Preferential regrowth of tumours by stem cells expressing low antigen levels may therefore occur following antibody mediated drug cytotoxicity and emphasises the need for a 'cocktail' of monoclonal antibodies recognising non associated antigens. There was no positive correlation between expression of the three monoclonal antibodies for any of the cells studied. In fact, many cells which expressed low levels of CEA expressed very high levels of the Y haptenic blood group antigen.

Prolonged culture of the cells produced further antigenic variation. Although in the majority of clones this was due to a reduction in expression of all 3 antigens, several clones showed enhanced binding of C14/1/46/10 monoclonal antibody. A similar drift in cell surface properties of tumour cells *in vitro* was noted by Neri *et al.*, (1981) and is a problem when designing model systems for testing the cytotoxicity of drug-antibody conjugates.

All of the cell lines had abnormal karyotypes which were reflected in their DNA indices obtained by flow cytometry. A similar observation was described by Drewinko *et al.* (1984), who subdivided colorectal cell lines on their DNA content, morphological differentiation, tumourigenicity, growth in soft agar and in tissue culture and on their levels of secreted CEA. Cell lines C146 and C170 could be ascribed by similar criteria to group I and C168 to group II. Growth of cells with abnormal DNA contents from tumours C146 and C170, which had diploid DNA contents, was particularly interesting. The clonogenic cells may all be aneuploid but comprise such a small proportion of the whole tumour they were undetected by the initial flow cytometric analysis. The aneuploid stem cells may preferentially grow in soft agar or short term tissue culture may induce chromosomal abnormalities. However, although 168 cells were aneuploid their DNA index was less than observed for the primary tumour suggesting that tissue culture may also cause chromosome segregation.

As all of the clonogenic cells grew readily in athymic mice without alteration in antigenicity and at least one monoclonal antibody, 791T/36, localised efficiently within these xenografts, they should be a good model for studying *in vivo* effects of drug-antibody conjugates.

It was surprising that an adenoma grew so readily in soft agar and that the isolated clones grew as vigorously as the carcinoma derived clones. However, the ability of these cells to form tumours in athymic mice and their aneuploid DNA content suggests that the adenoma contained a small malignant foci undetected histologically.

In the context of *in vivo* localisation of monoclonal antibodies it has already been established that radiolabelled 791T/36 antibody localises in primary or metastatic human colorectal tumours (Farrands *et al.*, 1982; Armitage *et al.*, 1984), but the intratumoral site of production and/or expression of the antigen was previously poorly defined. As immunohistology with 791T/36 antibody showed reactions primarily in tumour stromal elements and pseudoacini contents rather than malignant cell surfaces (Armitage *et al.*, 1983). The present studies have demonstrated that the antigen defined by 791T/36 monoclonal antibody is produced by cells grown *in vitro* from colorectal tumours. In addition the growth of these clonogenic populations in nude mice provides xenograft tumour models for experimental studies on localisation of 791T/36 antibody for diagnostic or therapeutic application.

This study therefore demonstrated that colorectal tumours with high growth potential *in vitro* express a range of tumour associated antigens, several at higher levels than observed in the primary tumour. Careful selection of monoclonal antibody 'cocktails' should therefore allow antibody mediated drug cytotoxicity to be effective at eradicating rapidly dividing tumour cells.

These studies were supported by the Cancer Research Campaign, UK. The skilful technical assistance of Mrs R.A. Marksman and Mr O. Roberts is gratefully acknowledged.

### References

ARMITAGE, N.C., PIMM, M.V., BALDWIN, R.W. & HARDCASTLE, J.D. (1983). The pattern of antigen distribution in colorectal tumours defined by monoclonal antibodies. *Br. J. Surg.*, **70**, 691.

ARMITAGE, N.C., PERKINS, A.C., PIMM, M.V., FARRANDS, P.A., BALDWIN, R.W. & HARDCASTLE, J.D. (1984). The localisation of an antitumour monoclonal antibody (791T/36) in gastrointestinal tumours. *Br. J. Surg.*, **71**, 407.

BRATTAIN, M.G., LEVINE, A.E., CHAKRABARTY, S., YEOMAN, L.C., WILLSON, J.K.V. & LONG, B. (1984). Heterogeneity of human colon carcinoma. *Cancer Met. Rev.*, **3**, 177.

BROWN, A., FEIZI, T., GOOI, H.C., EMBLETON, M.J., PICARD, J.K. & BALDWIN, R.W. (1983). A monoclonal antibody against human colonic adenoma recognises a difucosylated Type-2 blood group chains. *Bioscience Rep.*, **3**, 163.

BROWN, A., ELLIS, I.O., EMBLETON, M.J., BALDWIN, R.W., TURNER, D.R. & HARDCASTLE, J.D. (1984). Immunohistochemical localization of Y hapten and the structurally related H-type-2 blood group antigen on large bowel tumours and normal adult tissues. *Int. J. Cancer*, **33**, 727.

DEXTER, D.L., SPREMULLI, E.N., FLIGIEL, Z., BARBOSA, J.A., VOGEL, R., VAN VOORHEES, A. & CALABRESI, P. (1981). Heterogeneity of cancer cells from a single human colon carcinoma. *Am. J. Med.*, **71**, 949.

DREWINKO, B., YANG, L.Y., LEIBOVITZ, A., BARLOGIE, B., LUTZ, D., JANSSON, B., STRAGAND, J.J. & TRUJILLO, J.M. (1984). Cellular discriminants for a biological classification of human colon carcinoma. *Cancer Res.*, **44**, 4241.

DURRANT, L.G., PARKER, M., KENWORTHY, N. & TAYLOR, G.M. (1984). Characterization of a human mouse T cell hybridoma and identification of a clone secreting and binding interleukin-2. *Immunology*, **52**, 117.

DURRANT, L.G., ROBINS, R.A., ARMITAGE, N.C., BROWN, A., BALDWIN, R.W. & HARDCASTLE, J.D. (1985). Association of antigen expression and DNA ploidy in colorectal tumor. *Cancer Res.*, (in press).

EMBLETON, M.J., GUNN, B., BYERS, V.S. & BALDWIN, R.W. (1981). Antitumour reactions of monoclonal antibody against a human osteogenic sarcoma cell line. *Br. J. Cancer*, **43**, 582.

EDWARDS, P.A.W. (1985). Heterogeneous expression of cell surface antigens in normal epithelia and their tumours, revealed by monoclonal antibodies. *Br. J. Cancer*, **51**, 149.

FARRANDS, P.A., PERKINS, A.C., PIMM, M.V., HARDY, J.G., BALDWIN, R.W. & HARDCASTLE, J.D. (1982). Radioimmunodetection of human colorectal cancers using an anti-tumour monoclonal antibody. *Lancet*, **ii**, 397.

FINAN, P.J., GRANT, R.M., DE MATTOS, C. & 4 others. (1982). Immunohistochemical techniques in the early screening of monoclonal antibodies to human colonic epithelium. *Br. J. Cancer*, **64**, 9.

GALLEGO, J., PRICE, M.R. & BALDWIN, R.W. (1984). Preparation of four daunomycin-monoclonal antibody 791T/36 conjugates with antitumour activity. *Int. J. Cancer*, **33**, 737.

GARNETT, M.C., EMBLETON, M.J., JACOBS, E., & BALDWIN, R.W. (1983). Preparation and properties of a drug-carrier-antibody conjugate showing selective antibody-directed cytotoxicity *in vitro*. *Int. J. Cancer*, **31**, 661.

HAMBURGER, A.W. (1981). Use of *in vitro* tests in predictive cancer chemotherapy. *J. Natl. Cancer Inst.*, **66**, 981.

MOERTEL, C.G. (1973). Cancer of the large bowel. In *Cancer Medicine*, Trei and Holland (eds) p. 1597. Lea and Febiger: Philadelphia.

NERI, A. & NICHOLSON, G.L. (1981). Phenotypic drift of metastatic and cell surface properties of mammary adenocarcinoma cell clones during growth *in vitro*. *Int. J. Cancer*, **28**, 731.

PELHAM, J.M., GRAY, J.D., FLANNERY, G.R., PIMM, M.V. & BALDWIN, R.W. (1983). Interferon conjugation to human osteogenic sarcoma monoclonal antibody 791T/36. *Cancer Immunol. Immunother.*, **15**, 210.

PERKINS, A.C., PIMM, M.V. & BIRCH, M.K. (1985). The preparation and characterisation of $^{111}$In-labelled 791T/36 monoclonal antibody for tumour immunoscintigraphy. *Eur. J. Nucl. Med.*, (in press).

PRICE, M.R., CAMPBELL, D.G., ROBINS, R.A. & BALDWIN, R.W. (1983). Characteristics of a cell surface antigen defined by an anti-human osteogenic sarcoma monoclonal antibody. *Eur. J. Cancer Clin. Oncol.*, **19**, 81.

PRICE, M.R., BROWN, A., ARMITAGE, N.C.M. & BALDWIN, R.W. (1985). Application of a micro radioimmunoassay to analysis of monoclonal antibody defined epitopes on antigen preparations from human colonic cancer. *IRCS Med. Sci.*, **13**, 366.

PRIMUS, F.J. & GOLDENBERG, D.M. (1982). Functional histopathology of cancer: a review of immunoenzyme histochemistry. *Methods Cancer Res.*, **20**, 139.

ROE, R., ROBINS, R.A., LAXTON, R.R. & BALDWIN, R.W. (1985). Kinetics of divalent monoclonal antibody binding to tumour cell surface antigens using flow cytometry: standardization and mathematical analysis. *Mol. Immunol.*, **22**, 11.

ROSENTHAL, K.L., TOMPKINS, W.A.F., FRANK, G.L., McCULLOCH, P. & RAWLS, W.E. (1977). Variants of a human colon adenocarcinoma cell line which differs in morphology and carcinomenbryonic antigen production. *Cancer Res.*, **37**, 4024.

SILVERBERG, E. (1983). Cancer statistics. *C. A. Canc. J. Clin.*, **33**, 9.

STEEL, G.G. (1977). Growth kinetics of tumours. p. 217. Oxford University Press: London.

STOWER, M.J. & HARDCASTLE, J.D. (1985). The results of 1115 patients with colorectal cancer treated over an 8 year period in one hospital. *Eur. J. Surgical Oncol.*, **11**, 119.